# Osteoporosis Complications in Crohn's Disease Patients: Factors, Pathogenesis, and Treatment Outlines

**DOI:** 10.7759/cureus.20564

**Published:** 2021-12-21

**Authors:** Yaqot N Baban, Christopher M Edicheria, Joseph Joseph, Parneet Kaur, Jihan A Mostafa

**Affiliations:** 1 Research, California Institute of Behavioral Neurosciences and Psychology, Fairfield, USA; 2 Internal Medicine, California Institute of Behavioral Neurosciences and Psychology, Fairfield, USA; 3 Medicine, California Institute of Behavioral Neurosciences and Psychology, Fairfield, USA; 4 Faculty Member, California Institute of Behavioral Neurosciences and Psychology, Fairfield, USA

**Keywords:** crohn's disease, osteoporosis, ospteopenia, cytokines, steroids

## Abstract

The causes of osteoporosis in Crohn’s disease (CD) are multifactorial; cytokines, steroids, and vitamin deficiency all have an essential role. It is imperative to distinguish the factors that contribute to bone resorption, potentially increasing the risk of low bone mineral density (BMD), osteoporosis, and fracture. However, the pathogenicity of osteoporosis associated with CD remains unclear. Although osteoporosis treatment may vary between bisphosphonate and corticosteroid, infliximab's efficacy, when combined with immune modulators, suppresses both CD symptoms and osteoporosis progression. In this review, we aim to understand the present pathogenicity of osteoporosis, including the factors pro-inflammatory cytokines, chronic steroid use, and malnutrition, developing osteoporosis in a different pathological way, and to assist the treatment lines implying a positive outcome of osteoporosis in CD patients.

Osteoporosis is considered to be one of the early complications of CD where early detection can prevent osteoporosis progression. This can be done by utilizing dual-energy X-ray-absorptiometry (DEXA) to evaluate the Z-score and treat the existing factors that have a role in the progression of osteoporosis in CD patients.

## Introduction and background

Crohn’s disease (CD) is the inflammation of the gastrointestinal tract with skipped lesions from the mouth to the anus, with or without involving the rectum. It is one of the spectrums of inflammatory bowel disease (IBD). The most common symptoms of CD include abdominal pain, fever, diarrhea with the passing of blood or mucous, or both [1]. The causes of CD vary; it is a complex interplay of genetic factors, immune dysregulation, and bacterial dysbiosis [2]. It is associated with intestinal complications like bleeding from ulcers, fistulas, strictures, and colon cancer. In contrast, extra-intestinal complications affect different aspects of the body, like the ophthalmological, dermatological, pulmonary, and bone systems, which eventually lead to either osteopenia or osteoporosis. In IBD, the prevalence of osteopenia and osteoporosis is 22-77% and 17-41%, respectively [3]. Vazquez et al. stated that data regarding fracture prevalence in IBD patients has yet to be clarified, and Bernstein et al. concluded that the prevalence of fracture in IBD patients is 40% higher than in the healthy population [4,5]. Histologically, osteoporosis is characterized by thinning and fragmentation of the trabecular bone, which is irreversible when it is far advanced. Due to the nature of CD, patients may initially develop osteopenia, resulting in decreased bone formation and mineralization. If osteopenia is left untreated, it will progress into osteoporosis [6]. Typically, bones in healthy adults are in a state of balance between bone formation (osteoblast) and bone resorption (osteoclast). There are biochemical markers of bone turnover, such as deoxypyridinoline (DPD) and cross-linked N-telopeptides of type collagen type 1 (Ntx), associated with bone resorption. However, osteocalcin and bone-specific alkaline phosphatase (BSAP) are bone formation markers. These markers have been used in the process of bone demineralization in patients with CD [7]. In CD, higher levels of either DPD or Ntx are present due to bone resorption and reduced bone formation [8].

The inflammatory response of the immune system has a direct impact on osteoporosis in CD. The Pro-inflammatory cytokines (IL-1 beta, IL-6, IL-17, TNF-alpha, INF-gamma), and other TNF-related cytokines like receptor of activated nuclear factor-kappa B (RANK) and its ligand (RANKL) or osteoprotegerin (OPG), which is an osteoblast derived soluble decoy receptor. Those altogether accelerate the progress of osteopenia and osteoporosis, as this imbalance will increase bone resorption and decrease bone formation [9]. 

Additionally, glucocorticoids have a critical impact on the progression of osteoporosis in CD. Unfortunately, long-term use of steroids can contribute to an acceleration in bone loss as it affects the OPG-RANKL-RANK pathway [2]. Klaus et al. reported that male patients with CD also presented with complaints of low BMD and osteoporosis. Androgen is a natural hormone in the body that converts into estrogen and plays a vital role in decreasing bone resorption; corticosteroid use, in addition to the inflammatory process of CD, decreases dehydroepiandrosterone sulfate (DHEA), leading to a defect in androgen levels, reducing estrogen; thus, it affects bone remodeling, causing low BMD and osteoporosis as the normal process of estrogen increases OPG, which is a bone resorption marker [9].

Malnutrition is another factor in osteoporosis. CD leads to malabsorption of dietary nutrients such as iron, vitamin B12, vitamin C, vitamin K, vitamin D, phosphorous, zinc, and potassium [10]. Vitamin D and calcium play an essential role in bone metabolism and homeostasis. Simultaneously, patients with CD may not tolerate dairy products such as milk, cheese, and yogurt, leading to decreased calcium intake [11]; the entire process of bone formation is disrupted, leading to osteoporosis [12]. Moreover, studies show that vitamin B12 and folate deficiencies can increase homocysteine levels, which play a role in bone demineralization [13]. Also, vitamin K is the main factor in the carboxylation of osteocalcin, so the malabsorption of vitamin K showed a rise in nonfunctional osteocalcin levels, affecting bone mineralization [14].

To select the most appropriate treatment to stop the progression of osteoporosis in CD, we should treat the factors that contribute to the development of osteoporosis. Up to date, the most effective treatments that decelerate osteoporosis progression and CD symptoms are steroid-sparing medications that increase the BMD by ~3% [15,16]. Meanwhile, the combination of vitamin D, calcium, and bisphosphonates has a great effect on osteoporosis by suppressing osteoclast activity [17]. Although the long-term use of corticosteroids is the leading cause of osteoporosis, a study showed a decrease in BMD by ~3% per year. The alternative is the short-term use of steroids only at the flare-ups or locally acting steroids that reduce the systematic absorption [16,18]. 

As soon as the diagnosis of CD is confirmed by clinicians, the patients should undergo evaluation for osteoporosis by DEXA scan according to the American Gastroenterology Association (AGA) guidelines. As osteoporosis is an early extra-intestinal complication of CD, treatments and supplements should be started once CD is diagnosed to prevent complications such as fragility fractures [19]. The study aims to conduct a literature review by collecting studies from previously published papers about CD and osteoporosis, focusing on the main factors that lead to osteoporosis. Then go over the studies that support treatment regimens used in patients with CD that suppress the progression of osteoporosis and prevent fractures in this population.

## Review

The pathophysiology of osteoporosis in CD

The bone remodeling process is important for normal bone formation without excessive bone formation. This process is controlled by the integrity of the OPG-RANKL-RANK axis. OPG normally inhibits RANKL from binding to RANK, and it acts as natural apoptosis of the amateur osteoclasts' differentiation into mature osteoclasts [20]. This alteration can be determined by measuring biological markers in the serum and urine, but they could also be undetected [21]. The most common markers are carboxylated osteocalcin, bone-specific alkaline phosphatase, and procollagen peptides that indicate bone formation and they are detected in the serum, while bone resorption markers, one collagen cross-linked N-telopeptide (NTx), hydroxyproline, pyridinoline, and deoxypyridinoline, can be detected in the urine, and tartrate-resistant acid phosphate is detected in the serum [22].

In CD, the inflammatory process mediated by the cytokines (IL-1, IL-6, IL-17, TNF-alpha, and INF-gamma) will alter the RANKL function as the activated T-cell itself increases RANKL expression. This alteration will lead to an increase in bone resorption and destruction [20]. Turk et al. discussed that serum osteocalcin was low, and NTx was high compared to healthy participants, which was correlated to inflammation triggered by cytokines. The same study illustrated the effect of corticosteroids on the bone and showed a decrease in osteocalcin level, indicating bone resorption and osteoporosis development [22]. Another aspect of osteoporosis in CD is that malnutrition occurs due to malabsorption. Bastos et al. reported that most patients would complain of a decrease in dairy intake due to intolerance, which leads to a low level of vitamin D and calcium. The decline in vitamin D and calcium causes the secretion of parathyroid hormone (PTH) to restore normal calcium levels. However, PTH increases RANKL expression and inhibits OPG, which eventually causes bone resorption [23].

According to AGA guidelines on osteoporosis, and with all risk factors related to low BMD, osteopenia, and osteoporosis in CD patients, those patients should undergo screening for bone density at the early stage of CD. The study by Wagnon et al. supported the importance of bone scanning by conducting a study comparing two groups of clinicians and their awareness of the AGA guidelines regarding the diagnosis and treatment of metabolic bone disease in IBD patients. The first group of clinicians were aware of the guidelines and followed them as AGA recommended, while the second group were either unaware or did not follow the AGA guidelines. The study showed that the first group of 87% (109/126) was aware and followed AGA guidelines. 91% of their patients were evaluated and screened for bone metabolic disease with a DEXA scan, and it was found that 95% of the patients had osteoporosis. The second group was unaware or did not follow the guidelines with 99% (131/132), but 75% of their patients were screened for osteoporosis with a DEXA scan, and 72% were positive for osteoporosis. In addition, they addressed the most common reasons why most clinicians do not evaluate osteoporosis in CD patients. As a result, they found the center of interest of 42% of clinicians were the CD symptoms only, and 30% of them considered that the diagnosis of osteoporosis should be evaluated and managed by other providers. They recommended that those clinicians refer their patients to other providers if they are not willing to consider the evaluation and treatment of osteoporosis [19].

The DEXA scan uses the Z-score to determine if the patients have osteoporosis or osteopenia. A Z-score of greater than −1 indicates normal bone density, a Z-score of −1 to −2.5 indicates osteopenia, and a Z-score of less than −2.5 indicates osteoporosis [24]. Researchers recommend that, as in these studies, DEXA scans be used in the process of assessing and evaluating the early presentation of patients with CD symptoms in order to detect bone resorption. As a result, the patients will have a better outcome regarding bone health [19,24].

Factors that contribute to osteoporosis in CD patients

**Figure 1:**
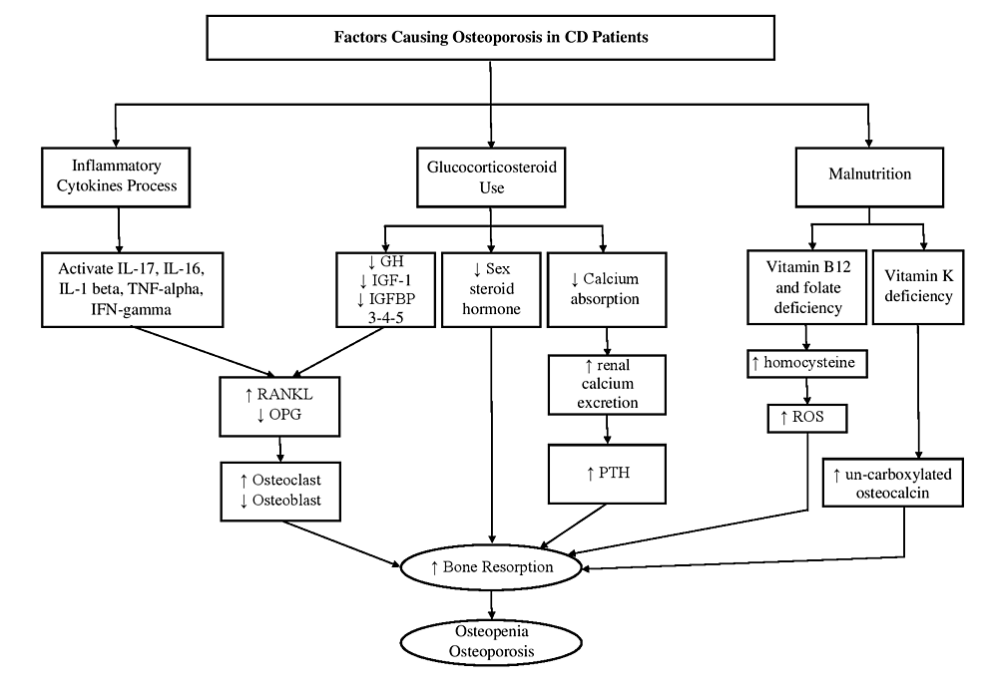
Pathophysiology of the factors that related to the development of osteopenia and osteoporosis as a consequence in CD patients. GH, growth hormone; IGF-1, insulin growth factor-1; IGFBP 3-4-5, insulin growth factor binding protein 3-4-5; RANKL, receptor activator of NF- Kb; OPG, osteoprotegerin.

As the incidence of low BMD, osteopenia and osteoporosis are some of the extra-intestinal complications in CD patients, many factors have been related to the imbalance of bone homeostasis with different pathways. The etiology remains unclear, but it is distributed among the inflammatory cytokines, corticosteroid use, and malnutrition, which all have a significant role in developing bone turnover and bone remodeling imbalances. The flowchart in Figure 1 illustrates the related factors of pathophysiology in CD.

Inflammatory cytokines

Several studies have been conducted in different populations, including Polish and Iranian [25,26]. These studies have found that the leading cause of CD is the cytokines' pro-inflammatory processes due to immune system activation. The most common cytokines that play a significant role in CD are IL-1beta, IL-6, IL-17, TNF-alpha, and IFN-gamma. The specific cytokine that activates the process of osteoporosis is TNF-alpha, as it provokes the OPG-RANKL-RANK axis. It was also found that IL-6 has a direct effect on the OPG-RANKL by provoking osteoclastogenesis. Krela-Kazmierczak et al. observed in a study conducted on 37 patients and demonstrated the prevalence of osteoporosis in the lumbar spine (L2-L4) by 18.92% and 13.51% in the neck. These findings took place because of the direct impact of IL-6 on bone turnover. This process is accomplished by inhibiting OPG function and provoking the bone resorption process by activating the axis of RANKL binding to RANK [26]. Another study conducted by Ali et al. discussed that IL-6, IL-17, IL-1 beta, and TNF-alpha have another pathway in bone resorption by direct RANKL activation [3].

The dual activities of the cytokines in provoking CD symptoms, the destruction of bone integrity, and developing osteoporosis are still unclear, but as soon as the patient is diagnosed with CD, a treatment to subside the action of the cytokines should be started to put the patient in remission from the CD symptoms and stop the progression of osteoporosis.

Glucocorticosteroids

Another factor that contributes to the progression of osteoporosis is glucocorticosteroids. Steroids have been used in the treatment of inflammatory bowel disease as they are the most effective treatment that induces remission in patients with flare-ups by rapid healing of the intestinal mucosa [27]. Lane et al. and Swanson et al. discussed the pathology of steroids-induced osteoporosis as it works as an anti-anabolic and decrease the effects of growth hormone (GH), insulin growth factor-1 (IGF-1), and insulin growth factor binding protein 3-4-5 (IGFBP3-4-5), which leads to an increase in RANKL and a decrease in OPG. It eventually increases osteoclasts and bone resorption [27,28]. In a review, Vavricka et al. discussed a group of four categories of patients with moderate to severe symptoms of CD, whom they had treated with different doses, routes of administration, duration, and treatment responses. The first group was on oral corticosteroid of 40-60 mg for 30 days, the second group was on a high dose of intravenous corticosteroids for 7-10 days, the third group was patients who did not respond to oral or intravenous corticosteroids, and the fourth group was patients who became dependent on corticosteroids as the relief of their symptoms was maintained with daily 10-30 mg oral steroids. They came to the result that 50% of patients on glucocorticosteroids become either dependent or resistant, and those found in the fourth group were shown to be at a higher risk of developing and worsening osteoporosis due to using steroids as maintenance medication [29]. As calcium is one of the main bone components, Rubin et al. evaluated the interaction between glucocorticosteroid treatment and calcium metabolism and revealed that glucocorticosteroid decreases calcium absorption and increases renal calcium secretion. As a result, the parathyroid function increased and led to secondary hyperparathyroidism and bone resorption [30].

As glucocorticosteroids dramatically induce bone destruction and decrease bone formation in different pathological ways, Canalis et al. have found that men on glucocorticosteroids develop bone resorption due to reduced sex steroid hormone production, and hypogonadism has been confirmed by increasing deoxypyridinoline excretion, which is a bone resorption marker [31]. Thus, glucocorticosteroids with the effect of bone destruction can be used as small doses of short duration in flare-ups to prevent osteoporosis progression or development. 

Malnutrition

The inflamed and injured intestinal mucosa predisposes the patient to nutrient and vitamin deficiencies as the causes are multifactorial, such as malabsorption, chronic diarrhea, chronic use of medications, and low oral intake [32]. Coqueiro et al. conducted a study of 60 patients with CD who were 18 years of age or older. The study involved the evaluation of body mass index and waist circumference and performing a DEXA scan of the whole body, specifically the lumbar vertebrae and femoral neck. They concluded that patients with a prolonged gastrointestinal condition such as a CD along with nutritional factor deficiency are at an increased risk of developing osteopenia and osteoporosis [10]. Andreassen et al. reported that many studies and publications were conducted on vitamin D and calcium deficiency and metabolic bone disease in CD, while few studies pointed to vitamin K deficiency. They explained that vitamin K works on osteocalcin's carboxylation, which has a role in bone-building by coupling calcium with osteocalcin and delivering it to the bone [33]. In addition, Schoon et al. demonstrated that CD patients had lower serum vitamin K levels than the control group (p < 0.01). It was recognized that CD patients had elevated un-carboxylated osteocalcin circulating, which was related to low vitamin K levels (p < 0.05) [14].

Another nutritional factor is vitamin B12 and folate deficiency. When these two nutrient levels are low, homocysteine levels elevate; the latter acts as an indicator showing that vitamin B12 and folate deficiencies increase osteoporosis. One study by Kim et al. showed that homocysteine has a role in decreasing bone density and osteoporosis. Hyperhomocysteinaemia increases bone apoptosis by increasing reactive oxygen species (ROS) [34]. Furthermore, Koh et al. study showed that homocysteinemia's role in osteoporosis was due to provoking osteoclast formation activity via the p38 MAPK pathways and ROS system activation [35]. According to CD patients' nutritional status, more studies are needed to outline the importance of nutrient deficiency in bone demineralization. 

Recommended treatment outlines to decrease the process of osteoporosis in CD patients

One of the main complications of CD in the long term is osteoporosis. Different studies were conducted to find the best treatment. Each treatment has a different mechanism for subsiding the progression of osteoporosis in CD patients. 

Steroid sparing medications

Infliximab is anti-TNF-alpha. By blocking TNF-alpha, T-cells will be suppressed, increasing the level of OPG and stopping the disturbance of the OPG-RANKL-RANK axis, improving bone formation and reducing bone resorption [15]. A study discussed how the maintenance of infliximab intake will improve bone density; Mauro et al. conducted a study with a 22-month follow-up and found an increase in lumbar bone density of about 14% and a 7% increase in body weight; this study not only proved that infliximab improved the bone density but also improved the patients’ wellbeing by upgrading the bodyweight and decreasing the gastrointestinal symptoms [36]. When CD was diagnosed and the patient was started on corticosteroids, the risk of osteoporosis increased as the patients themselves had developed low BMD due to the process of CD. In the ACCENTI randomized trial by Hanauer et al., two groups of CD patients were given different Infliximab dosages, receiving 5 mg/kg and 10 mg/kg, respectively; 25% of the first group and 35% of the second group showed a meticulous response to discontinuing corticosteroid therapy and remaining in remission [37].

A review by Cushing et al. compared immune modulators (methotrexate and thiopurine) and biological drugs (infliximab, adalimumab, and vedolizumab), showing a good impact regarding maintenance and flare-up management of CD symptoms and decreasing the long-term complications of corticosteroid use as well as the effect of CD on bone destruction and slowing down the osteoporosis progression. Moreover, treating patients with immune modulator drugs as monotherapy drugs has a limited impact compared to using biologics alone or combining biologics with thiopurine [38]. Another study by Colombel et al. was conducted and appraised on the combination of biologics and thiopurines. The survey was conducted on 508 patients with moderate to severe CD, 170 on thiopurine, 169 on anti-TNF, and 169 on thiopurine plus anti-TNF. The results at 26 weeks of follow-up of steroid-free treatment manifest: patients on thiopurine monotherapy were in remission by 30%, those on anti- TNF monotherapy were in remission by 44% (p-value of 0.006), while patients on the combination of thiopurine and anti-TNF therapy were in remission by 56% (p-value of <0.001). This study clarified that using thiopurines such as azathioprine as an adding drug to anti-TNF such as infliximab will give the patients a better outcome regarding the disease remission and decrease the intestinal and extra-intestinal complications [39]. A study by Krajcovicova et al. supported the importance of the combination of anti-TNF and azathioprine as both will positively affect the BMD and decrease the risk of osteopenia and osteoporosis. Using these drugs will reduce the need for corticosteroids as they have a negative impact on the bone [29].

Consequently, infliximab alone or in combination with immune modulator drugs should be considered as an early line in the therapy of patients with CD to prevent bone resorption and maintain remission.

Bisphosphonates (1-10 mg) with calcium (500-800 mg) and vitamin D (400-1000 mg) supplementation

The mechanism of action of bisphosphonates is by inhibiting osteoclast-mediated bone resorption as it inhibits the activity of mature osteoclasts and their proliferation, differentiation, and access to the bone precursor. For many years, bisphosphonates were added to the treatment of patients with CD who were taking corticosteroids and supported with supplements of calcium and vitamin D. In a study by Haderslev et al. conducted on patients taking 10 mg of alendronate with 400 IU of vitamin D and 500 mg of calcium daily, reported increased bone density [40]. Sebba et al. pointed out that most patients were non-compliant with the treatment regimen of oral bisphosphonate due to the gastric upset associated with alendronate. They planned to administer a new regimen of 1 mg of intravenous ibandronate every three months; consequently, the change in drug administration revealed better outcomes associated with increased bone density and no gastric upset [41]. Von Tirpitz et al. compared three different groups treated with an osteo-anabolic agent (sodium fluoride) 25 mg twice a day orally, ibandronate 1 mg every three months intravenously, and vitamin D3 (1000 IU) and calcium citrate (800 mg). These groups were followed for 12 and 27 months. As a result, the bone density increased by 4.1% after one year and 5.7% in 2.25 years for those treated with sodium fluoride. Meanwhile, in the group treated with ibandronate, the bone density increased by 3.5% after one year and 5.4% after 2.25 years. They also proved supplements of calcium and vitamin D improved bone density and weight gain when they were combined with bisphosphonate and were statistically significant with a p-value of <0.001 [42].

Related to treatment and prevention with bisphosphonates in CD patients, Soo et al. compared the first, second, and third generations of bisphosphonates and found that the first-generation (etidronate) did not improve bone density. In contrast, second and third-generation (alendronate) and (risedronate) improved bone density after one year of follow-up with vitamin D and calcium supplements [43]. According to the discussed studies, patients with or without steroid use in CD should be provided with bisphosphonate and calcium with vitamin D supplements, helping to increase BMD and suppress the progression of osteoporosis. Patients may experience possible side effects such as gastric upset due to bisphosphonates and calcium supplements. For this reason, they have to be educated about the benefits of taking these medications to prevent further worsening of osteoporosis.

Corticosteroids

Due to the flare-ups that patients with CD encountered, the most effective treatment path is corticosteroid administration, as it has a rapid positive effect on mucosal healing for those with mild to moderate symptoms. A review by Bernstein et al. discussed the benefits of corticosteroids in the remission of flare-ups and the side effects of the long-term use of steroids may affect bone health and progress to low BMD and osteoporosis. However, they reported a conflict about which process affects bone health, either the glucocorticosteroid itself or the inflammatory cytokines' provocation in glucocorticosteroid users [18].

At this point, researchers were conducting research to find another type of steroid that could be used safely and with minimal side effects on bone. A review by Vavricka et al. revealed a non-systemic steroid called budesonide as a substitute for prednisolone. It is recommended for mild to moderate symptoms with ileum and proximal colon involvement as it is cleared hepatically with low systemic absorption and less bone destruction [29]. A study by Sherlock et al. observed two groups of patients. The first group was treated with systemic steroids and the remission rate was 52-89%, and the second group was treated with budesonide and the remission rate was 51-60%. These results determined that budesonide is analogous to systemic steroids in inducing the remission of CD patients’ flare-ups [44]. Despite all the factors that cause worsening of osteoporosis in CD patients, each element has a treatment regimen that will suppress both the CD symptoms and the osteoporosis progression and provide a good outcome for the patients.

Limitation: The limitation of our review is that the research tool was only the PubMed database; thus, we might not have covered all factors that evolve the osteoporosis in CD patients. Moreover, this review contained different study designs, populations, sample sizes, and studies of 10 years and more as we did not apply the inclusion and exclusion criteria to our review. 

## Conclusions

Osteoporosis is the most common extra-intestinal complication of Crohn’s disease. We reviewed a variety of factors that contribute to low bone density and the development of osteoporosis in CD, such as the inflammatory response of the cytokines by alteration of the OPG-RANKL-RANK axis and the increase in bone resorption that is detected by increasing the level of the resorption markers DPD and NTx. Another factor is that chronic use of the steroid pathway will affect the androgen level that will decrease DHEA. As a result, there will be a low estrogen level that contributes to the development of osteoporosis. Lastly, malabsorption of vitamin D, vitamin K, and calcium will increase the incidence of bone demineralization through different mechanisms such as increased PTH, homocysteinemia, and activation of the ROS pathway.

Researchers propose assessing bone density loss using a DEXA scan and Z-score to determine the level of osteoporosis. The DEXA scan will help the healthcare community and primary care providers precisely, as they are the first to be contacted by patients with CD symptoms. By incorporating the DEXA scan into routine diagnostic tools and diagnosing osteopenia or osteoporosis as soon as the disease manifests itself, these efforts prevent further damage to the bone and give the patients the healthiest quality of life during the relapse and remission of the CD symptoms. As low bone density and osteoporosis were diagnosed, treatment with different resources started, such as immunosuppressive and biological drugs, which play a role in controlling the CD symptoms and increasing bone density. The second line is bisphosphonate with vitamin D and calcium, which improves bone resorption by inhibiting the osteoclast process and preventing further bone destruction. Finally, the local steroid budesonide has been found to have a less systematic effect while also treating CD flare-ups. Future studies need to be conducted so that these questions can be answered. As CD is an immune-related condition, are the family members of CD patients screened for CD? More prospective cohort studies are needed for those at risk of CD to help determine if there are any other causes related to osteoporosis in CD patients.
